# Supplementary material for: Structure and efflux mechanism of the yeast pleiotropic drug resistance transporter Pdr5
Source: Nat Commun. 2021 Sep 6;12:5254. doi: 10.1038/s41467-021-25574-8 (PMC8421411; doi:10.1038/s41467-021-25574-8)
Supplement: Supplementary file 1 — Supplementary Information [file 41467_2021_25574_MOESM1_ESM.pdf]

SUPPLEMENTARY INFORMATION

Structure and efflux mechanism of the yeast pleiotropic drug resistance transporter Pdr5

Andrzej Harris[1], Manuel Wagner[2,a], Dijun Du[1,b], Stefanie Raschka[2], Lea-Marie Nentwig[2], Holger Gohlke[3,4,5,6], Sander H. J. Smits[2,7], Ben F. Luisi[1]*, Lutz Schmitt[2]*

[1]: Department of Biochemistry, University of Cambridge, 80 Tennis Court Road, Cambridge, CB2 1GA, United Kingdom
[2]: Institute of Biochemistry, Heinrich Heine University Düsseldorf, Universitätsstraße 1, 40225 Düsseldorf, Germany
[3]: Institute of Pharmaceutical and Medicinal Pharmacy, Heinrich Heine University Düsseldorf, Universitätsstraße 1, 40225 Düsseldorf, Germany
[4]: John von Neumann Institute for Computing (NIC), Jülich Supercomputing Centre (JSC), Forschungszentrum Jülich GmbH, 52425 Jülich, Germany
[5]: Institute of Biological Information Processing (IBI-7: Structural Biochemistry), Forschungszentrum Jülich GmbH, 52425 Jülich, Germany
[6]: Institute of Bio- and Geosciences (IBG-4: Bioinformatics), Forschungszentrum Jülich GmbH, 52425 Jülich, Germany
[7]: Center for Structural Studies, Heinrich Heine University Düsseldorf, Universitätsstraße 1, 40225 Düsseldorf, Germany

[a]: present address: Medac GmbH, Theatherstraße 6, 22880 Wedel, Germany
[b]: present address: School of Life Sciences and Technology, ShanghaiTech University, 393 Middle Huaxia Road, Pudong, Shanghai, China

*: to whom correspondence should be addressed:

Ben F. Luisi
Department of Biochemistry
University of Cambridge
80 Tennis Court Road
Cambridge CB2 1GA, United Kingdom
phone: 00441223766029
email: bfl20@cam.ac.uk

Lutz Schmitt
Institute of Biochemistry
Heinrich Heine University Düsseldorf
Universitätsstraße 1
40225 Düsseldorf, Germany
phone: 00492118110773
email: lutz.schmitt@hhu.de

These authors contributed equally: Andrzej Harris, Manuel Wagner, Dijun Du

Supplementary Figures

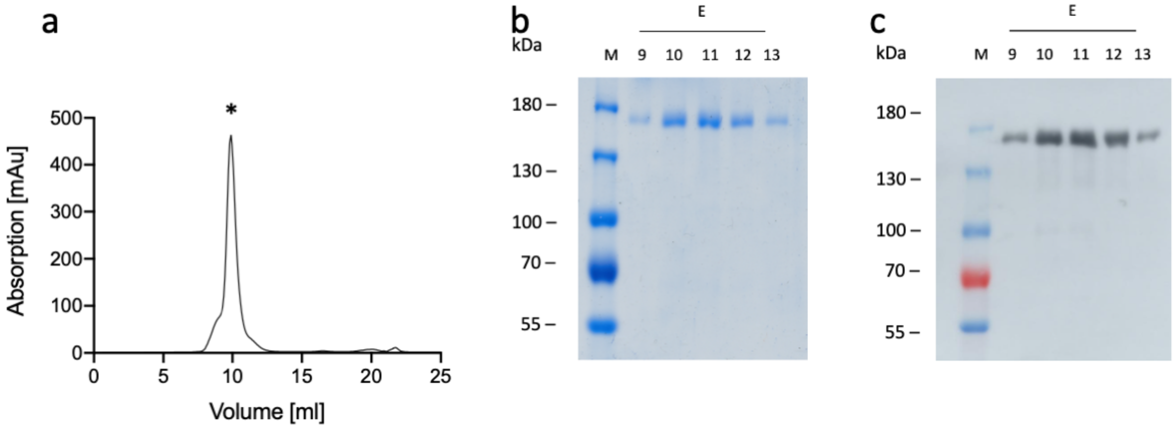

Supplementary Figure 1. Purification of Pdr5. a Size-exclusion chromatogram of Pdr5 solubilised in *trans*-PCC-α-M, as described in *Materials and Methods* section. Pdr5 elution is marked by an asterisk. **b** Coomassie-blue stained SDS-PAGE of purified Pdr5 (single repeat). **c** Western blot of the SDS-PAGE using a polyclonal Pdr5 antibody (single repeat). Molecular weight standards are given to the left of the SDS-PAGE and the Western blot. Source data are provided as a Source Data file.

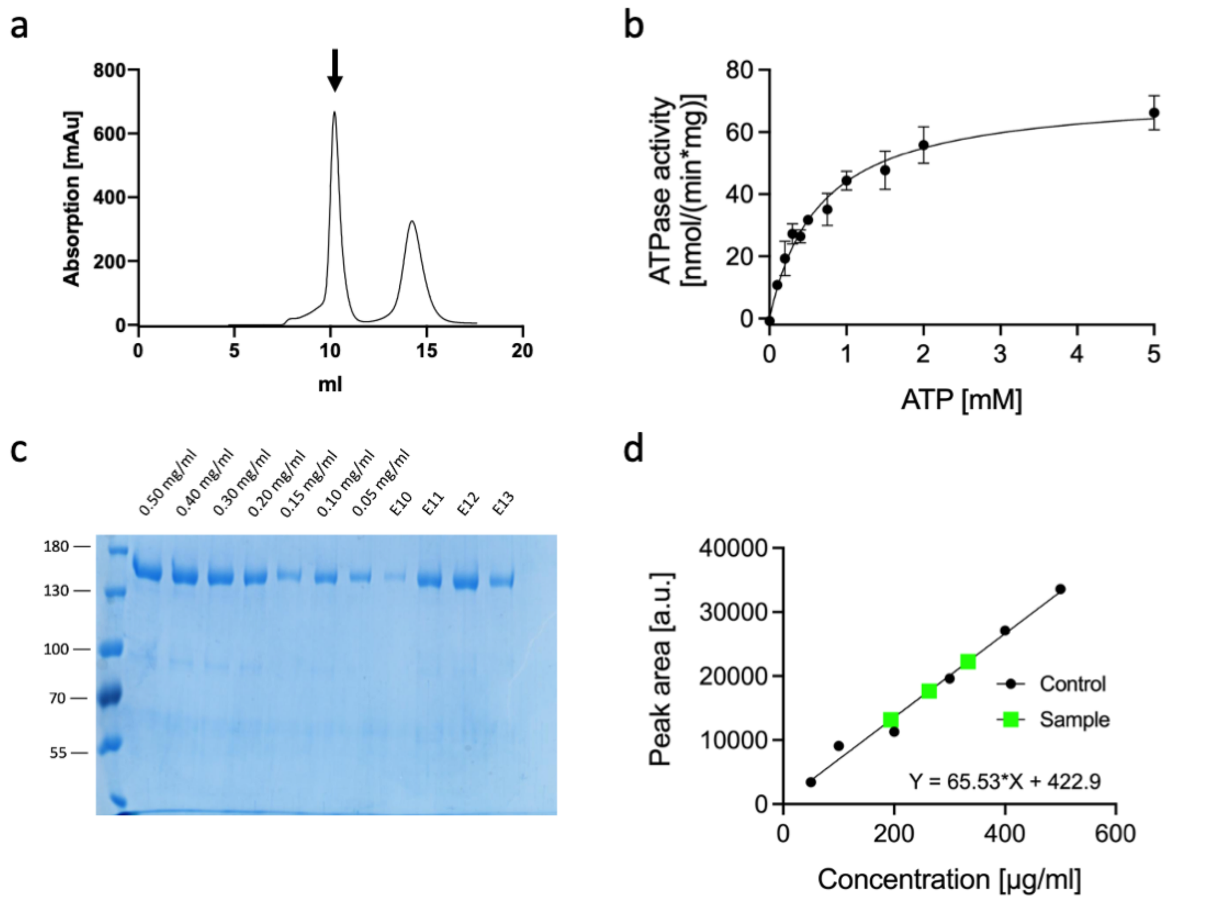

Supplementary Figure 2. Reconstitution of Pdr5 into peptidics. a Size-exclusion chromatogram of Pdr5 reconstituted into peptidisc, as described in *Materials and Methods* section. The peak of reconstituted Pdr5 is indicated by an arrow. **b** Pdr5 specific ATPase activity of reconstituted Pdr5. Error bars present mean ± standard error of three independent experiments. **c** Coomassie-blue stained SDS-PAGE of Pdr5 samples of known concentration and elution fractions of the reconstitution experiment (single repeat). **d** Calibration line of the samples of Pdr5 of known concentration (black circles) and the three elution fractions of the reconstitution experiment (green squares, E11 to E13 from left to right). The equation of the linear regression is also provided. Source data are provided as a Source Data file.

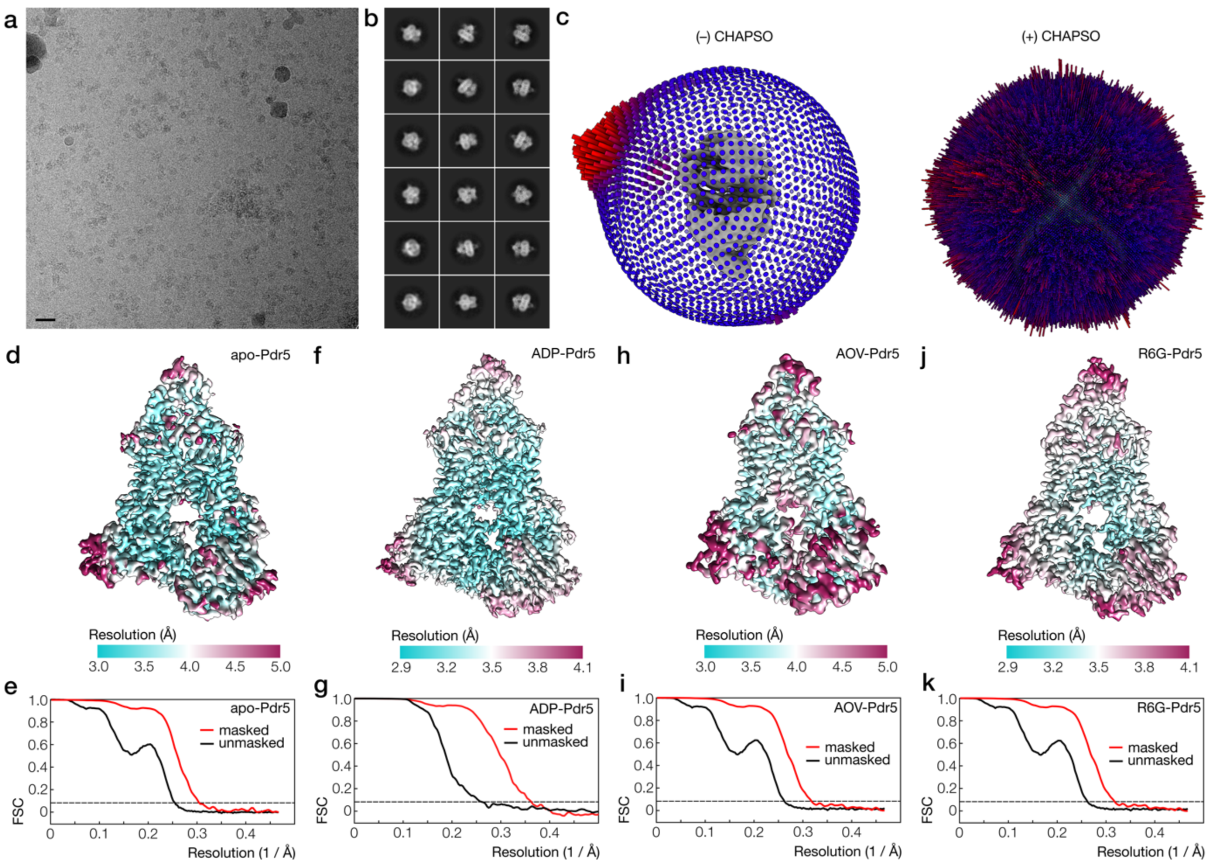

Supplementary Figure 3. Cryo-EM imaging of Pdr5. a Cryo-EM micrograph (selected from a set of 3,415) of Pdr5/peptidisc particles in vitrified ice (ADP-Pdr5 sample). Scale bar is 300 Å. **b** Averaged images of the particles, obtained in reference-free 2D classification. **c** Angular distribution of particle orientations in Pdr5 samples prepared with and without the addition of CHAPSO. **d** Cryo-EM map of apo-Pdr5, coloured by local resolution (see key). **e** Gold-standard FSC curves showing the correlation between the half-datasets in the unmasked (black line) and masked (red line) maps. The overall resolution was judged using the $FSC_{0.143}$ threshold (dashed line). **f**–**k** Cryo-EM maps and FSC curves for the other Pdr5 reconstruction, analogous to **d**–**e**. See also: Supplementary Table 1.
Abbreviations: FSC, Fourier-shell correlation.

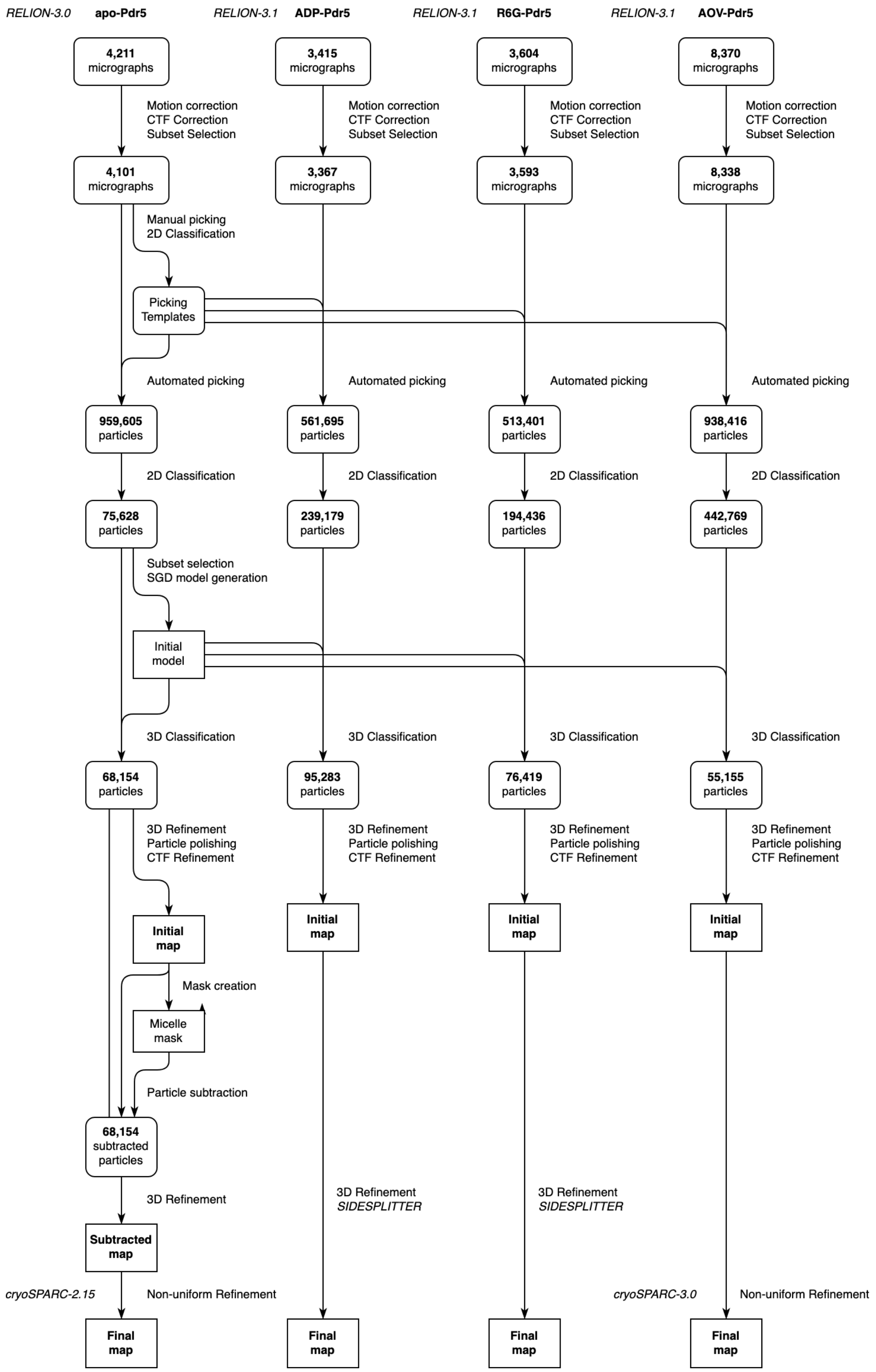

Supplementary Figure 4. Cryo-EM processing workflow for Pdr5 datasets. The diagram depicts schematically the steps in the computational analysis of the cryo-EM datasets that were collected in this study and used to reconstruct the four Pdr5 maps: apo-Pdr5, ADP-Pdr5, R6G-Pdr5 and AOV-Pdr5. Software packages, programs and individual procedures are listed, together with micrograph and particle numbers. Additional information is found in *Materials and Methods* section.

```
        10         20         30         40         50         60         70         80         90        100
                                    H1
MPEAKLNNNV NDVTSYSSAS SSTENAADLH NYNGFDEHTE ARIQKLARTL TAQSMQNSTQ SAPNKSDAQS IFSSGVEGVN PIFSDPEAPG YDPKLDPNSE
                                 NBD1

       110        120        130        140        150        160        170        180        190        200
  H2                                                   H3
NFSSAAWVKN MAHLSAADPD FYKPYSLGCA WKNLSASGAS ADVAYQSTVV NIPYKILKSG LRKFQRSKET NTFQILKPMD GCLNPGELLV VLGRPGSGCT
                                             (LD1)                                                   WA

       210        220        230        240        250        260        270        280        290        300
H4                        H5                              H6                    H7
TLLKSISSNT HGFDLGADTK ISYSGYSGDD IKKHFRGEVV YNAEADVHLP HLTVFETLVT VARLKTPQNR IKGVDRESYA NHLAEVAMAT YGLSHTRNTK
                                              Q

       310        320        330        340        350        360        370        380        390        400
            H8                              H9                              H10                           H11
VGNDIVRGVS GGERKRVSIA EVSICGSKFQ CWDNATRGLD SATALEFIRA LKTQADISNT SATVAIYQCS QDAYDLFNKV CVLDDGYQIY YGPADKAKKY
        SM                      WB        DL                              H

       410        420        430        440        450        460        470        480        490        500
              H12                  H13        H14                                   H15
FEDMGYVCPS RQTTADFLTS VTSPSERTLN KDMLKKGIHI PQTPKEMNDY WVKSPNYKEL MKEVDQRLLN DDEASREAIK EAHIAKQSKR ARPSSPYTVS
                                                                                                      TMD1

       510        520        530        540        550        560        570        580        590        600
TH1a             TH1b                                 TH2                                                TH3
YMMQVKYLLI RNMWRLRNNI GFTLFMILGN CSMALILGSM FFKIMKKGDT STFYFRGSAM FFAILFNAFS SLLEIFSLYE ARPITEKHRT YSLYHPSADA

       610        620        630        640        650        660        670        680        690        700
                                TH4                             TH5a                                     TH5b
FASVLSEIPS KLIIAVCFNI IFYFLVDFRR NGGVFFFYLL INIVAVFSMS HLFRCVGSLT KTLSEAMVPA SMLLLALSMY TGFAIPKKKI LRWSKWIWYI

       710        720        730        740        750        760        770        780        790        800
  TH5c                                                   H16                   TH6
NPLAYLFESL LINEFHGIKF PCAEYVPRGP AYANISSTES VCTVVGAVPG QDYVLGDDFI RGTYQYYHKD KWRGFGIGMA YVVFFFFVYL FLCEYNEGAK
                (PD1)                                                                                 (LD2)

       810        820        830        840        850        860        870        880        890        900
         H17
QKGEILVFPR SIVKRMKKRG VLTEKNANDP ENVGERSDLS SDRKMLQESS EEESDTYGEI GLSKSEAIFH WRNLCYEVQI KAETRRILNN VDGWVKPGTL
                                                                  NBD2

       910        920        930        940        950        960        970        980        990       1000
           H18                                  H19            H20                 H21
TALMGASGAG KTTLLDCLAE RVTMGVITGD ILVNGIPRDK SFPRSIGYCQ QQDLHLKTAT VRESLRFSAY LRQPAEVSIE EKNRYVEEVI KILEMEKYAD
    WA                                                 Q

      1010       1020       1030       1040       1050       1060       1070       1080       1090       1100
            H22                                H23                             H24
AVVGVAGEGL NVEQRKRLTI GVELTAKPKL LVFLDEPTSG LDSQTAWSIC QLMKKLANHG QAILCTIHQP SAILMQEFDR LLFMQRGGKT VYFGDLGEGC
         SM                     WB        DL                              H

      1110       1120       1130       1140       1150       1160       1170       1180       1190       1200
H25                   H26                   H27                                              TM7a
KTMIDYFESH GAHKCPADAN PAEWMLEVVG AAPGSHANQD YYEVWRNSEE YRAVQSELDW MERELPKKGS ITAAEDKHEF SQSIIYQTKL VSIRLFQQYW
                                                                      TMD2

      1210       1220       1230       1240       1250       1260       1270       1280       1290       1300
  TM7b                             TM8                                                TM9a
RSPDYLWSKF ILTIFNQLFI GFTFFKAGTS LQGLQNQMLA VFMFTVIFNP ILQQYLPSFV QQRDLYEARE RPSRTFSWIS FIFAQIFVEV PWNILAGTIA

      1310       1320       1330       1340       1350       1360       1370       1380       1390       1400
            TM9b    TM10                                 TM11a                           TM11b       TM11c
YFIYYYPIGF YSNASAAGQL HERGALFWLF SCAFYVYVGS MGLLVISFNQ VAESAANLAS LLFTMSLSFC GVMTTPSAMP RFWIFMYRVS PLTYFIQALL

      1410       1420       1430       1440       1450       1460       1470       1480       1490       1500
                           H28                                  H29        TM12
AVGVANVDVK CADYELLEFT PPSGMTCGQY MEPYLQLAKT GYLTDENATD TCSFCQISTT NDYLANVNSF YSERWRNYGI FICYIAFNYI AGVFFYWLAR
      (PD2)

      1510
VPKKNGKLSK K
```

Supplementary Figure 5. Annotated amino-acid sequence of Pdr5. Indicated are conserved features of the nucleotide binding sites (black boxes) and positions of α-helices (coloured boxes). The domains of Pdr5 are indicated, and the helices are coloured according to the scheme used elsewhere in the paper. Grey residues indicate parts of the ADP-Pdr5 model that could not be resolved in the cryo-EM map.

Abbreviations: DL, D-loop; ECD, extracellular domain; H, H-loop; LD, linker domain; NBD, nucleotide-binding domain; Q, Q-loop; SM, signature motif; TH, transmembrane helix; TMD, transmembrane domain; WA, Walker A (or P-loop); WB, Walker B (or A-loop).

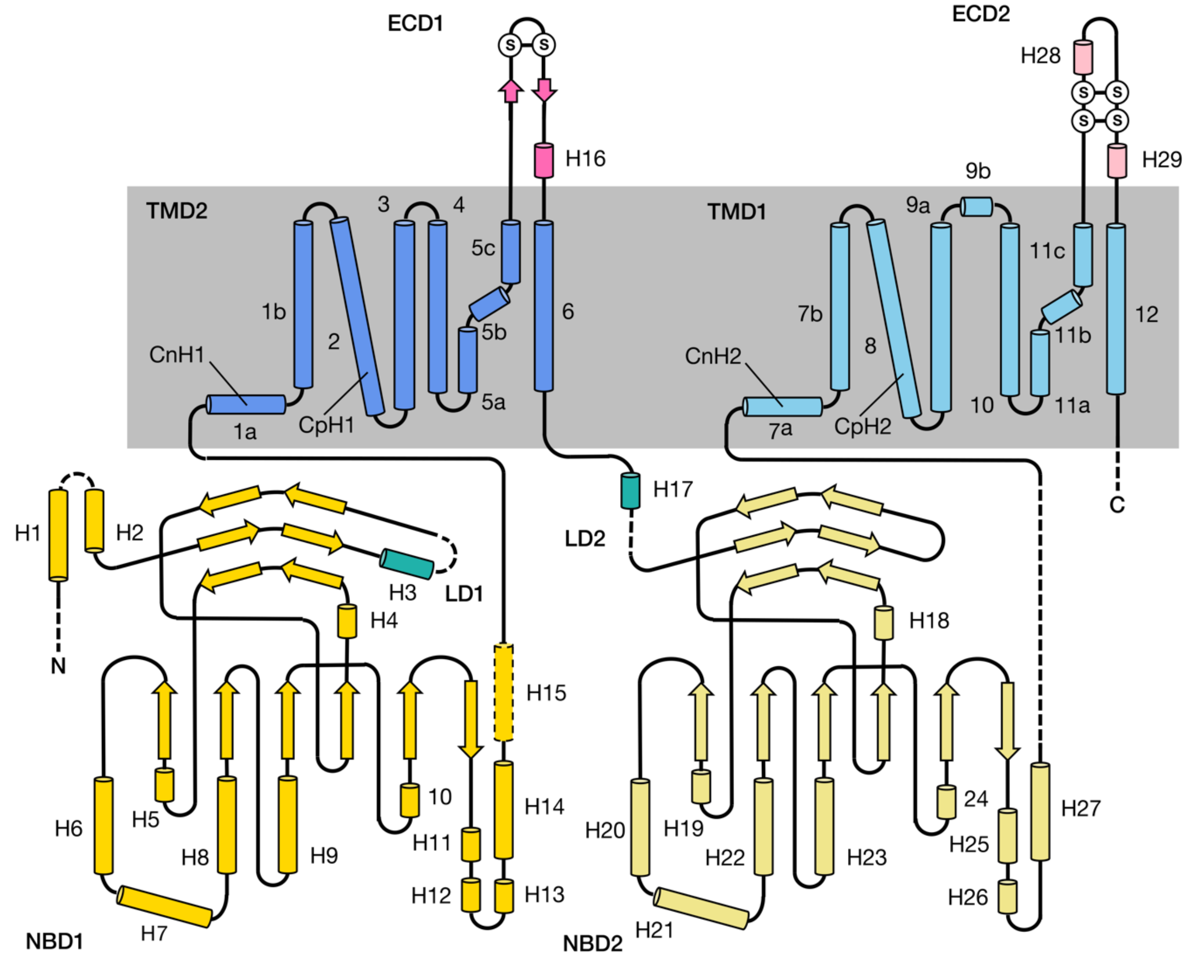

Supplementary Figure 6. Domain topology of Pdr5. A schematic representation of domain architecture of Pdr5 showing the arrangement of its secondary structural elements. Cylinders and arrows represent alpha-helices and beta-sheets. The elements on the diagram are not shown to scale, but their relative positioning in the two halves of the protein reflects the evolutionary relation of the transporter domains through a gene-duplication event. The colour scheme for domains is similar to the one used elsewhere in text. The dashed lines represent structure fragments that could not be resolved in the ADP-Pdr5 map (*cf*. Supplementary Figure 5). The circled letters "S" denote the position of disulphide bridges. According to the alternative nomenclature proposed for the PDR family[1], ECD1 and ECD2 are respectively EC3 and EC6.

Abbreviations: ECD, extracellular domain; LD, linker domain; NBD, nucleotide-binding domain; TMD, transmembrane domain.

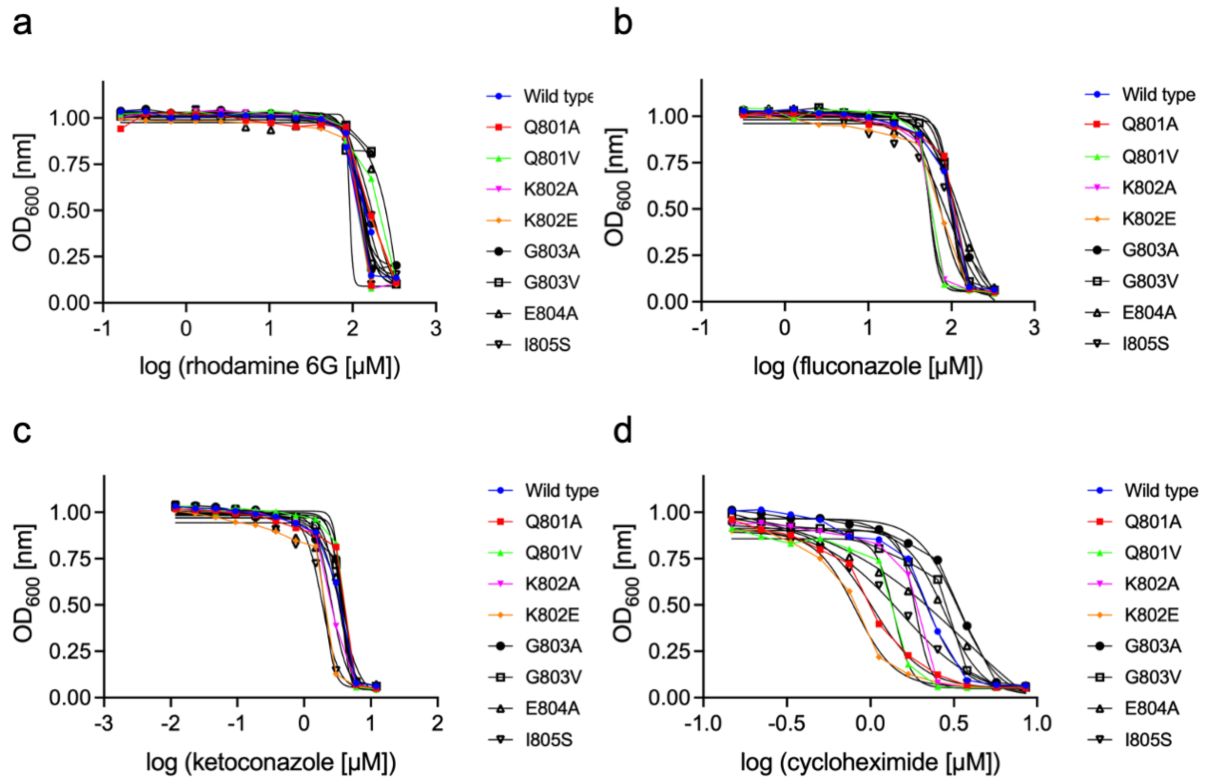

Supplementary Figure 7. Liquid drug assay for Pdr5 linker domain mutants. Yeast cells expressing Pdr5 (blue) or mutants in the linker domain (LD) were incubated for 48 hours at 30 °C with four different drugs at indicated concentrations. Subsequently, optical density at 600 nm (OD_{600}) was measured. Data were analysed using a log (inhibitor) vs response function implemented in Prism 9 (GraphPad Inc.). Experimental details of the assay are provided in the Methods section. The drugs investigated were **a** R6G, **b** fluconazole, **c** ketoconazole, and **d** cycloheximide. See also: Supplementary Table 3.

a

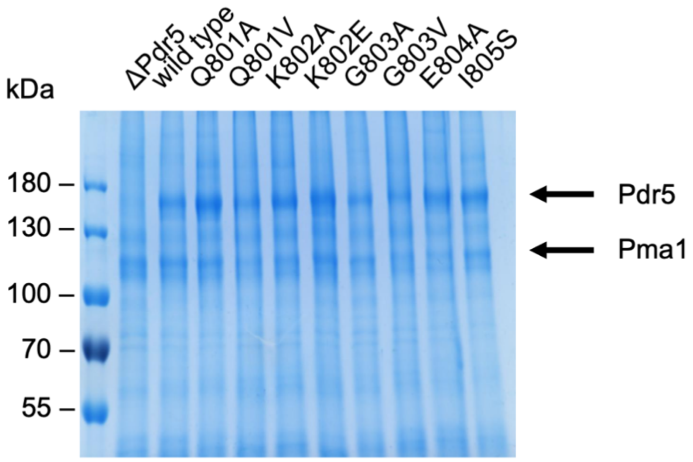

b

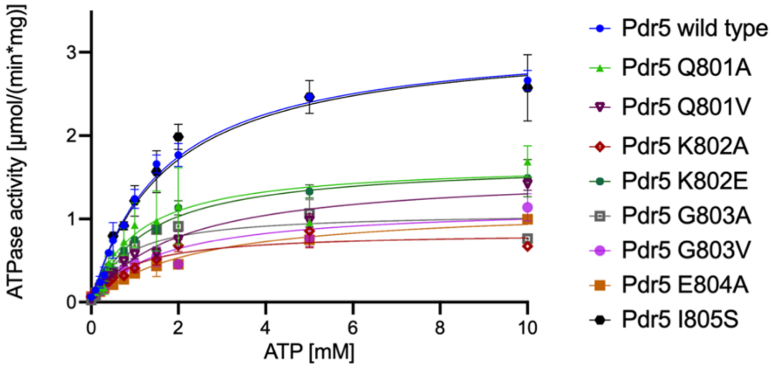

Supplementary Figure 8. ATPase activity of Pdr5 linker domain mutants in plasma membrane vesicles. a Coomassie-stained SDS-PAGE (single repeat) of plasma membrane vesicles containing wild-type Pdr5 and mutants in the linker domain (LD). The signals of Pdr5 and Pma1 (normalisation standard) are indicated with arrows. Densiometric analysis (Fiji) indicated differences in expression levels below 10%. The same result was obtained when taking the signal intensity of Pma1 into account. Therefore, no expression level correction was applied. **b** ATPase activity of Pdr5 reconstituted in plasma membrane vesicles. Shown are the kinetic curves for the ATP hydrolysis by wild-type protein and linker domain mutants. The experiment was performed using n=3 biologically independent samples examined over 3 independent experiments. Error bars in figure represent mean ± standard error. See also: Supplementary Table 4. Source data are provided as a Source Data file.

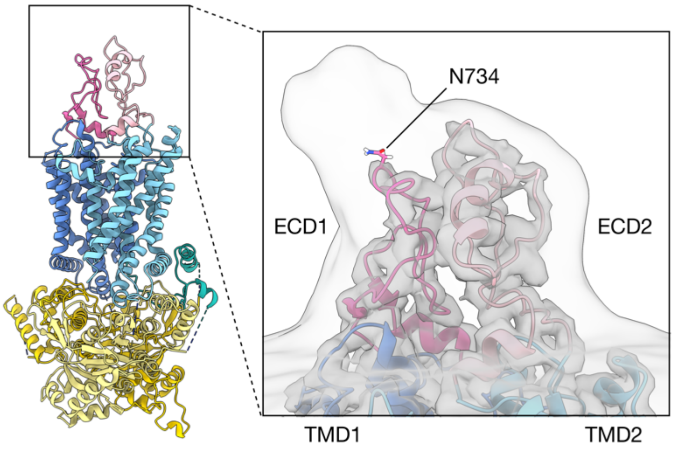

Supplementary Figure 9. Glycosylation of Pdr5. Figure shows the side view of the Pdr5 and, in inset, a putative glycosylation site on the extracellular domain of Pdr5. The two transparent surface representations of the apical portion of Pdr5 are from the same ADP-Pdr5 cryo-EM map, contoured at two different levels. The inner surface shows the Coulomb potential map immediately surrounding the protein; the outer is a noisier and Gaussian-filtered (2σ) version of the map, showing the protrusion around the asparagine residue (labelled), previously suggested to be glycosylated.
Abbreviations: ECD, extracellular domain; TMD, transmembrane domain.

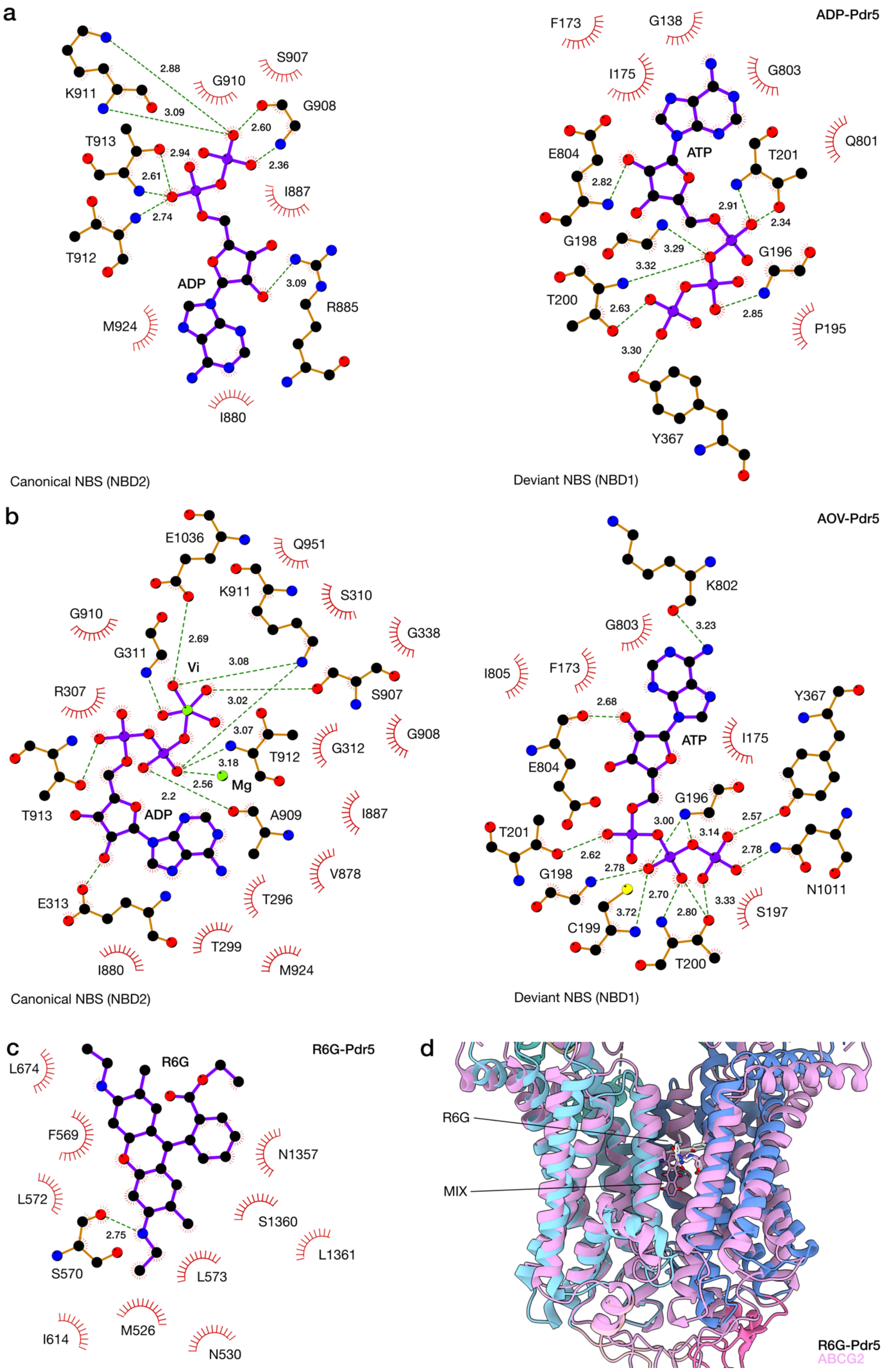
a
ADP-Pdr5
K911
2.88
3.09
G910
S907
G908
2.60
2.36
T913
2.94
2.61
2.74
I887
T912
ADP
3.09
R885
M924
I880
Canonical NBS (NBD2)
F173
G138
I175
G803
Q801
E804
ATP
T201
2.82
2.91
2.34
G198
3.29
G196
3.32
T200
2.63
2.85
P195
3.30
Y367
Deviant NBS (NBD1)
b
AOV-Pdr5
E1036
Q951
K911
S310
G910
G338
G311
2.69
Vi
3.08
R307
3.02
S907
3.07
G312
G908
T912
3.18
Mg
2.56
2.2
T913
ADP
A909
I887
V878
E313
T296
T299
I880
M924
Canonical NBS (NBD2)
K802
3.23
G803
I805
F173
2.68
Y367
ATP
I175
E804
G196
3.00
3.14
2.57
T201
2.62
2.78
2.78
G198
N1011
2.70
3.33
3.72
2.80
S197
C199
T200
Deviant NBS (NBD1)
c
R6G-Pdr5
L674
R6G
F569
N1357
L572
S1360
2.75
S570
L573
L1361
I614
M526
N530
d
R6G
MIX
R6G-Pdr5
ABCG2

Supplementary Figure 10. Details of protein-ligand interactions in Pdr5. Schematic representation of the network of molecular interactions between the nucleotides and the protein in the canonical and inactive nucleotide-binding sites of the **a** inward-facing ADP-Pdr5 and **b** the outward-facing AOV-Pdr5. The interactions between the transport substrate rhodamine 6G and the residues in the **c** substrate entry channel in the inward-facing R6G-Pdr5 are similarly depicted. Also shown is the superposition of cartoon models of R6G-Pdr5 and human multi-drug transporter ABCG2 (pink, PDB-ID: 6VXI) in complex with **d** the chemotherapy drug mitoxantrone. Interaction diagrams were prepared with LigPlot+[2]. Atoms are depicted as coloured balls (C, black; N, blue; O, red; P; purple; S, yellow; Mg, V, green), ligand covalent bonds are purple and protein bonds are brown. Dashed green lines represent hydrogen bonds or other electrostatic interactions, with inter-atom distances indicated in Å. The radiating arc symbol represents amino-acid residues involved in hydrophobic contacts. Abbreviations: MIX, mitoxantrone; R6G, rhodamine 6G.

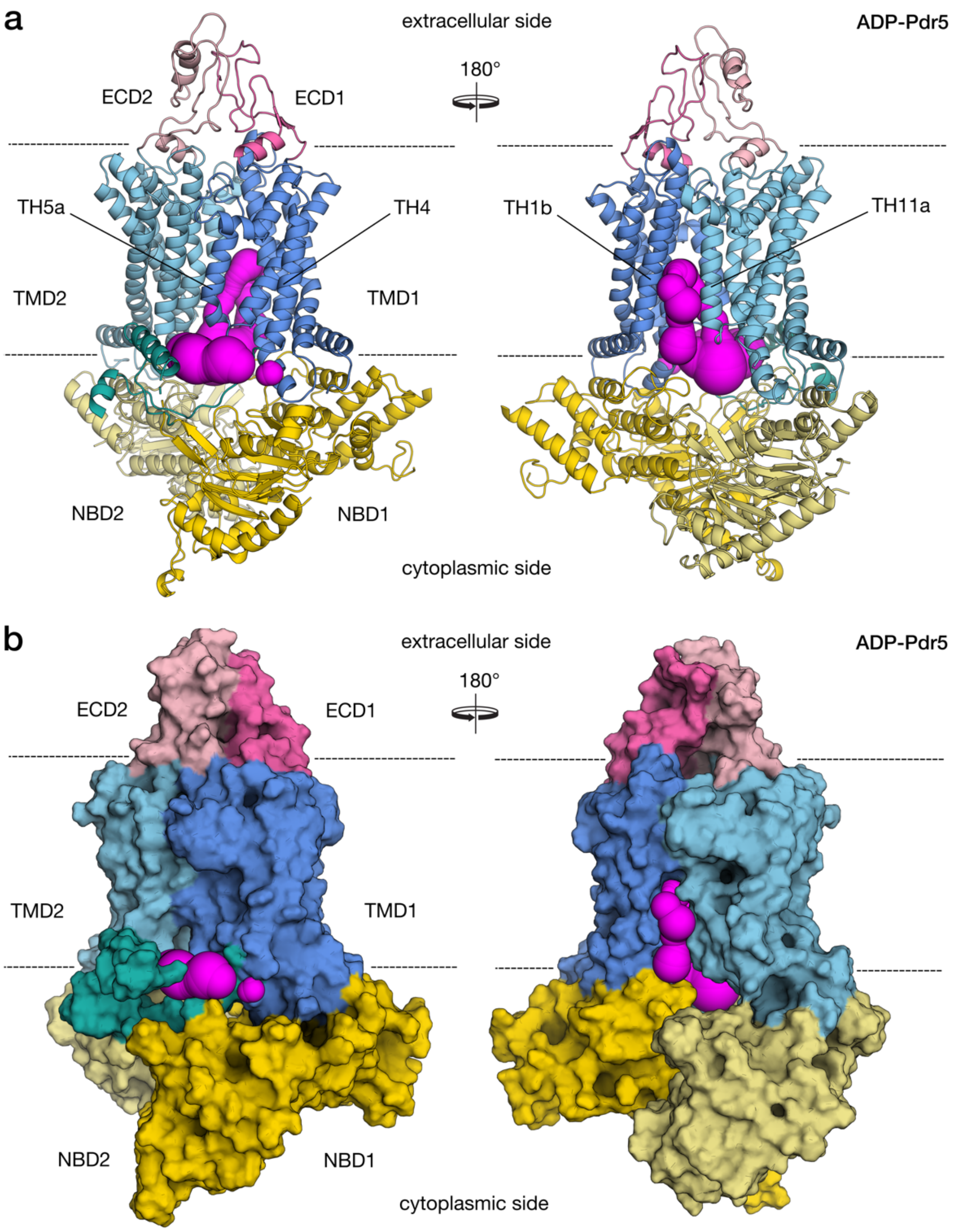

Supplementary Figure 11. Analysis of potential entry sites in the inward-facing conformation of Pdr5. a Cartoon representation of the inward-facing state and the present tunnels (magenta) as determined by CAVER-3.0[3]. **b** Surface representation of Pdr5. This analysis demonstrates that Pdr5 has only one membrane entry site between helices TH1b and TH11a, while the corresponding entrance on the opposite side is closed by TH4 and TH5a. The position of the membrane is indicated by dashed black lines.

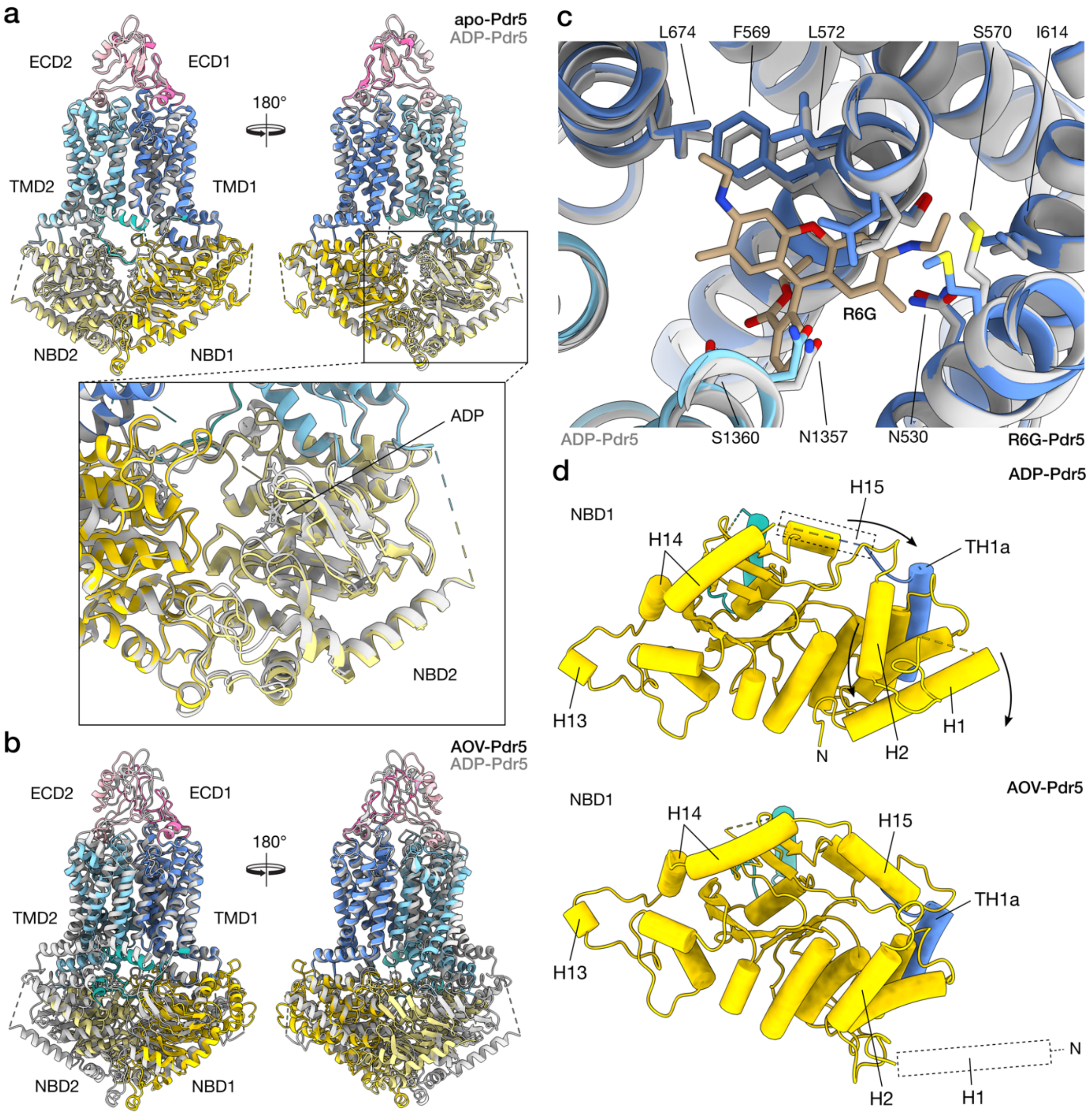

Supplementary Figure 12. Structural differences between Pdr5 models. Shown here are structural overlays of Pdr5 models in cartoon representation:) between apo-Pdr5 and ADP-Pdr5, with the inset showing details of the NBD2 domain around the catalytic NBS; **b** AOV-Pdr5 and ADP-Pdr5 (or outward-facing and inward-facing conformations); **c** between R6G-Pdr5 and ADP-Pdr5 in the region surrounding the rhodamine 6G transport substrate. In panels **a**–**c**, ADP-Pdr5 structure is used as a reference and is coloured grey. Other models follow the domain colouring scheme used elsewhere in the paper. **d** The diagram depicts the difference between the conformation of NBD1 domain of Pdr5 in outward- and inward-facing models (ADP-Pdr5 and AOV-Pdr5). In ADP-Pdr5, the N-terminal helices H1 and H2 are clearly resolved in the cryo-EM map, unlike helix H15, which did not adopt a discrete conformation. In AOV-Pdr5, H15 moves towards transmembrane helix TH1a, displacing H2 and H1, the latter of which is no longer visible in the map. Dashed lines denote the approximate position of unresolved helices, and arrows are an indication of movement.
Abbreviations: ECD, extracellular domain; NBD, nucleotide-binding domain; R6G, rhodamine 6G; TMD, transmembrane domain.

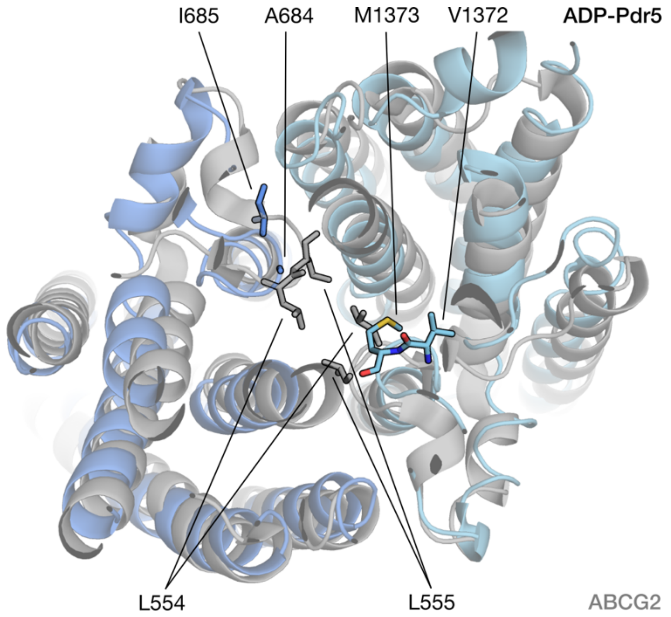

Supplementary Figure 13. Leucine gate of human ABCG2 and the corresponding region of Pdr5. Human ABCG2 (grey, PDB-ID: 6HCO [http://doi.org/10.2210/pdb6HCO/pdb]) and Pdr5 (coloured) were superimposed and the residues of ABCG2 forming the di-leucine gate (L554 and L555) are highlighted (ABCG is a homodimer). The corresponding residues of Pdr5 (A684/I685 and V1372/M1373) are also highlighted.

Supplementary Tables

Supplementary Table 1. Cryo-EM data collection and refinement statistics

Protein structure	apo-Pdr5	ADP-Pdr5	R6G-Pdr5	AOV-Pdr5
**Organism**	*S. cerevisiae*	*S. cerevisiae*	*S. cerevisiae*	*S. cerevisiae*
**PDB ID**	7P03	7P04	7P05	7P06
**EMDB ID**	EMD-13142	EMD-13143	EMD-13144	EMD-13145
**Data collection**				
**Microscope**	FEI Titan Krios	FEI Titan Krios	FEI Titan Krios	FEI Titan Krios
**Voltage (kV)**	300	300	300	300
**Detector**	K2 Summit	K3	K3	K3
**Mode**	counting	counting, super-resolution	counting, super-resolution	counting, super-resolution
**Nominal magnification**	130,000 ×	130,000 ×	130,000 ×	130,000 ×
**Pixel size (Å/pix)**	1.07	0.326	0.326	0.326
**Electron fluency, per frame (e^-/Å^2)**	1.452	0.96	0.97	0.98
**Electron fluency, total (e^-/Å^2)**	58.1	47.21	47.73	48.13
**Defocus range (µm), step (µm)**	–0.8 to –2.7, 0.3	–0.7 to –2.2, 0.3	–0.7 to –2.2, 0.3	–0.7 to –2.2, 0.3
**Exposure (s)**	12	1.31	1.31	1.31
**Frames**	40	49	49	49
**Number of micrographs**	4,211	3,415	3,604	8,370
**Reconstruction**				
**Software**	RELION-3.0 / cryoSPARC-2.15	RELION-3.1	RELION-3.1	RELION-3.1 / cryoSPARC-3.0
**Final number of particle images**	68,154	95,283	76,419	55,155
**Est. translational accuracy (pix)**	–	0.427	0.643	–
**Est. rotational accuracy (°)**	–	1.136	1.669	–
**Map resolution, $FSC_{0.143}$ (Å)**	3.45	2.85	3.13	3.75
**Map-sharpening B factor (Å^2)**	–102.9	–47.4	–55.0	–83.3
**Model composition**				
**Non-hydrogen atoms**	21,163	21,583	21,645	21,533
**Protein residues**	1,333	1,353	1,353	1,350
**Ligands (PDB)**	–	1 × ATP<br>1 × ADP	1 × ATP<br>1 × ADP<br>1 × R6G	1 × ATP<br>1 × AOV<br>1 × MG
**Refinement**				
**Software**	Phenix-1.18.2	Phenix-1.18.2	Phenix-1.18.2	Phenix-1.18.2
**Correlation coefficient, masked**	0.80	0.81	0.82	0.79
**Correlation coefficient, box**	0.67	0.60	0.66	0.52
**Model resolution, $FSC_{0.5}$ (Å)**	3.7	3.0	3.3	3.6
**Validation (proteins)**				
**MolProbity score**	1.71	1.62	1.67	1.91
**Clash score, all atoms**	6.14	7.14	7.16	9.39
**EMRinger score**	3.09	–	–	–
**Ramachandran plot statistics**				
**Favoured, overall (%)**	94.63	96.5	95.97	93.82
**Allowed, overall (%)**	5.37	3.5	4.03	6.81
**Outlier, overall (%)**	0.06	0	0	0
**R.m.s. deviations**				
**Bond length (Å)**	0.006	0.073	0.006	0.006
**Bond angle (°)**	0.678	0.711	0.551	0.703

Supplementary Table 2. Structural alignment of Pdr5 and related proteins.

Transporter	PDB-ID	R.m.s.d. vs. Pdr5 IF* [Å]	R.m.s.d. vs. Pdr5 OF** [Å]
**TarGH**	6jbh	14 (359)	13.9 (358)
**Wmz-Wzt / ATP**	6m96	9.2 (343)	8.8 (338)
**Wmz-Wzt / ATP-Mg^{2+}**	7k2t	13.4 (727)	10.5 (667)
**ABCA1**	5xjy	33.3 (762)	36.3 (808)
**ABGG2 OF****	6hbu	5.1 (1006)	2.8 (881)
**ABCG2 IF***	6hco	3.9 (929)	6.8 (988)
**ABCG5/G8**	5do7	4.3 (753)	6.6 (821)

Note: R.m.s.d. calculation considered only backbone atoms and was carried out in PyMOL (Schrödinger LLC). Numbers in parentheses indicate residue range of alignment. *: inward-facing; **: outward-facing.

Supplementary Table 3. Summary of the statistical analysis of the liquid drug assays.

Mutation	Drug			
	R6G	FC	KC	CH
**Q801A**	0.9976 (ns)	0.9817 (ns)	0.7321 (ns)	<0.0001 (****)
**Q801V**	0.9924 (ns)	<0.0001 (****)	0.9212 (ns)	<0.0001 (****)
**K802A**	0.9226 (ns)	<0.0001 (****)	0.1629 (ns)	0.0002 (***)
**K802E**	0.9968 (ns)	0.0032 (**)	0.0015 (**)	<0.0001 (****)
**G803A**	0.9998 (ns)	0.0468 (*)	0.8752 (ns)	0.9998 (ns)
**G803V**	0.2768 (ns)	0.9927 (ns)	0.9931 (ns)	0.4863 (ns)
**E804A**	0.0036 (**)	<0.0001 (****)	0.9974 (ns)	0.9112 (ns)
**I805S**	0.9998 (ns)	0.9994 (ns)	0.0002 (***)	<0.0001 (****)

Note: Analysis was carried out using a one-way ANOVA test in Prism 9 (GraphPad Inc.). ****: $p < 0.0001$, ***: $p < 0.0005$, **: $p < 0.005$, *: $p < 0.5$, not significant (ns): $p > 0.5$. See also: Supplementary Figure 7. Abbreviations: R6G, rhodamine 6G; FC, fluconazole; KC, ketoconazole; CH, cycloheximide.

Supplementary Table 4. Summary of the kinetic parameters of the ATPase activity of wild-type Pdr5 and the mutants of the linker domain.

Mutation	K_M [mM]	V_{max} [µmol min^{-1} mg^{-1}]	Difference with wild type	
			K_M	V_{max}
**Wild type**	1.7 ± 0.2	3.2 ± 0.1	–	–
**Q801A**	1.1 ± 0.4	1.7 ± 0.2	0.5913 (ns)	<0.0001 (****)
**Q801V**	2.0 ± 0.3	1.6 ± 0.1	0.98 (ns)	<0.0001 (****)
**K802A**	0.9 ± 0.2	0.8 ± 0.1	0.2786 (ns)	<0.0001 (****)
**K802E**	1.3 ± 0.2	1.7 ± 0.1	0.9078 (ns)	<0.0001 (****)
**G803A**	0.7 ± 0.1	1.1 ± 0.1	0.1016 (ns)	<0.0001 (****)
**G803V**	1.6 ± 0.4	1.2 ± 0.1	0.9997 (ns)	<0.0001 (****)
**E804A**	2.3 ± 0.4	1.2 ± 0.1	0.5913 (ns)	<0.0001 (****)
**I805S**	1.7 ± 0.3	3.2 ± 0.2	>0.9999 (ns)	>0.9999 (ns)

Note: Statistical analysis was caried out using a one-way ANOVA test (Prism 9, GraphPad Inc.) and revealed no significant differences (ns) in K_M, but highly significant differences for seven of the eight mutants (****, $p < 0.0001$). Errors report standard deviation of at least three independent experiments.

Supplementary Table 5. Selected mutations of Pdr5 with functional impact and possible structural explanation of the observed functional modulation of Pdr5.

Mutation	Location	Modulation of Pdr5 function	Reference	Explanation based on the structures
**E244G**	Q-loop of NBD1	Increased drug sensitivity	[4]	Interaction with D-loop of NBD1
**T257I**	H6	Alters drug specificity	Uniprot P33302	Not evident
**G302D**	Loop preceding the C-loop of NBD1 (X-loop)	Generalized drug resistance	Uniprot P33302	Stabilizes the conformation of the C-loop of NBD1
**S558Y**	TH2	Impaired transport activity	[5]	Interacts with the linker TH11a-TH11b
**S648F**	TH4	Alters drug specificity	Uniprot P33302	Forms a helical bundle with TH5c and TH6
**G905S**	Walker A of NBD2	Inactivates transport	Uniprot P33302	ATP binding / hydrolysis
**G908S**	Walker A of NBD2	Inactivates transport	Uniprot P33302	ATP binding / hydrolysis
**Q951G**	Q-loop of NBD2	Increased drug sensitivity	[4]	ATP binding / hydrolysis
**G1009C**	Loop preceding the C-loop of NBD2 (X-loop)	Generalized drug resistance	Uniprot P33302	Interaction with LD1 (QKGEIL)
**G1040D**	D-loop of NBD2	Alters drug specificity	Uniprot P33302	Impaired NBD-NBD communication
**D1042N**	D-loop of NBD2	Alters drug specificity	[6]	Impaired NBD-NBD communication
**S1048V**	H23	Alters drug specificity	Uniprot P33302	Not evident
**Y1311S**	TH9b	Alters drug specificity	Uniprot P33302	Cluster of Y residues (1301, 1305, 1306, 1311)
**S1360F**	TH11a	Alters drug specificity	[7]	Part of the R6G binding site
**S1386A**	TH11a	Creates bi-directional transport	[8]	On top of the R6G binding site
**T1393I**	TH11c	Alters drug specificity	Uniprot P33302	On top of the R6G binding site

Note: The multiple suppressor mutants of Pdr5, which have been described in the literature (see, for example[4,9]), have been excluded due to potential additive effects that cannot be predicted from the wild-type structure.

Supplementary References

1. Lamping, E. *et al.* Fungal PDR transporters: Phylogeny, topology, motifs and function. *Fungal Genetics and Biology* **47**, 127–142 (2010).
2. Laskowski, R. A. & Swindells, M. B. LigPlot+: Multiple Ligand–Protein Interaction Diagrams for Drug Discovery. *J. Chem. Inf. Model.* **51**, 2778–2786 (2011).
3. Chovancova, E. *et al.* CAVER 3.0: a tool for the analysis of transport pathways in dynamic protein structures. *PLoS Comput Biol* **8**, e1002708 (2012).
4. Ananthaswamy, N. *et al.* The signaling interface of the yeast multidrug transporter Pdr5 adopts a cis conformation, and there are functional overlap and equivalence of the deviant and canonical Q-loop residues. *Biochemistry* **49**, 4440–4449 (2010).
5. Sauna, Z. E. *et al.* Mutations Define Cross-talk between the N-terminal Nucleotide-binding Domain and Transmembrane Helix-2 of the Yeast Multidrug Transporter Pdr5. *J Biol Chem* **283**, 35010–35022 (2008).
6. Furman, C. *et al.* The Deviant ATP-binding Site of the Multidrug Efflux Pump Pdr5 Plays an Active Role in the Transport Cycle. *J. Biol. Chem.* **288**, 30420–30431 (2013).
7. Kueppers, P., Gupta, R. P., Stindt, J., Smits, S. H. J. & Schmitt, L. Functional Impact of a Single Mutation within the Transmembrane Domain of the Multidrug ABC Transporter Pdr5. *Biochemistry* **52**, 2184–2195 (2013).
8. Mehla, J. *et al.* Evidence for a molecular diode-based mechanism in a multispecific ATP-binding cassette (ABC) exporter: SER-1368 as a gatekeeping residue in the yeast multidrug transporter Pdr5. *J Biol Chem* **289**, 26597–26606 (2014).
9. Downes, M. T. *et al.* The Transmission Interface of the Saccharomyces cerevisiae Multidrug Transporter Pdr5: Val-656 Located in Intracellular Loop 2 Plays a Major Role in Drug Resistance. *Antimicrob Agents Chemother* **57**, 1025–1034 (2013).
