# Supplementary material for: Structure and efflux mechanism of the yeast pleiotropic drug resistance transporter Pdr5
Source: Nat Commun. 2021 Sep 6;12:5254. doi: 10.1038/s41467-021-25574-8 (PMC8421411; doi:10.1038/s41467-021-25574-8)
Supplement: Supplementary file 5 — Description of additional supplementary files [file 41467_2021_25574_MOESM5_ESM.docx]

Description of additional supplementary files

Title: Supplementary Movie 1

Description: Conformational changes in Pdr5

Title: Supplementary Movie 2

Description: Pdr5 transport substrate channel
